# Real-world treatment patterns and outcomes among patients initiating sequential regorafenib and trifluridine/tipiracil ± bevacizumab in patients with metastatic colorectal cancer in a US community setting (SEQRT2)

**DOI:** 10.3389/fonc.2025.1591245

**Published:** 2025-06-11

**Authors:** Tanios Bekaii-Saab, Ila Sruti, Junxin Shi, Wei Dai, Gregory Patton, Sreevalsa Appukkuttan, Brian Hocum, Arvind Katta, Svetlana Babajanyan, David Cosgrove

**Affiliations:** ^1^ Medical Oncology, Mayo Clinic Cancer Center, Phoenix, AZ, United States; ^2^ Real World Research, Ontada, Boston, MA, United States; ^3^ US Medical Affairs, Bayer Healthcare Pharmaceuticals Inc., Whippany, NJ, United States; ^4^ Compass Oncology, The US Oncology Network, Vancouver, WA, United States

**Keywords:** metastatic colorectal cancer, regorafenib, real world research, clinical outcomes, treatment patterns, sequencing, access

## Abstract

**Background:**

Regorafenib and trifluridine/tipiracil (FTD/TPI) ± bevacizumab are both indicated for patients diagnosed with metastatic colorectal cancer (mCRC) in the third line or later. However, in the absence of recommendations regarding preferred treatment order, our study aimed to improve the understanding of real-world optimal treatment sequences.

**Methods:**

This retrospective study assessed real-world outcomes and treatment patterns among mCRC patients who initiated sequential regorafenib and FTD/TPI ± bevacizumab between the first line and sixth line from September 2015 to November 2022 in The US Oncology Network. Patient and treatment characteristics were assessed descriptively overall and stratified by treatment order. The Kaplan–Meier methods were used for time-to-event endpoints, including real-world overall survival (rwOS), real-world progression-free survival (rwPFS), and real-world time to next treatment (rwTTNT) following sequence. Endpoints were also evaluated using Cox proportional hazards models.

**Results:**

This study examined 308 patients initiating sequential regorafenib and FTD/TPI, 156 patients initiating regorafenib first (R-T), and 152 patients initiating FTD/TPI first (T-R). Demographic and clinical characteristics were similar across cohorts. The population was predominantly male and had a mean age of 63 years and colon primary at diagnosis. The median rwOS was numerically longer among the R-T cohort compared to the T-R cohort (12.8 [11.2, 14.1] vs. 10.2 [8.8, 11.9] months). The median rwPFS was similar (3.4 [3.0, 3.6] vs. 3.4 [3.0, 3.7 months) for both the R-T and T-R cohorts. The median rwTTNT following sequence was numerically longer among the R-T cohort compared to the T-R cohort (9.3 [8.4, 10.3] vs. 8.6 [7.8, 9.4] months). Index treatment was not significantly associated with rwOS (Hazard Ratio (HR) = 1.2, p = 0.2), rwPFS (Hazard Ratio (HR) = 0.9, p = 0.4), or rwTTNT (Hazard Ratio (HR) = 1.1, p = 0.6).

**Conclusion:**

Treatment sequence numerically favored R-T and provided an additional survival benefit of 2.6 months in this cohort, although this was not statistically significant. Providing access to regorafenib and FTD/TPI ± bevacizumab is critical to maximizing patient benefit and optimizing patient care in advanced stages of mCRC.

## 1 Introduction

Colorectal cancer (CRC) is the second leading cause of cancer-related deaths in the United States (1). Approximately 152,810 new cases of CRC and 53,010 deaths were expected in 2024 in the United States (US) (2). Approximately 50%–60% of patients diagnosed with CRC develop metastases (3, 4).

Regorafenib and trifluridine/tipiracil (FTD/TPI), approved by the Food and Drug Administration (FDA) in 2012 and 2015, respectively, are both indicated for patients with metastatic CRC (mCRC) who have failed prior standard therapies, usually after at least two lines of therapy, and have been shown to extend overall survival (5, 6). National Comprehensive Cancer Network (NCCN) Clinical Practice Guidelines in Oncology (NCCN Guidelines^®^) do not indicate a preference for initial therapy (7). Although regorafenib or trifluridine/tipiracil have been found to have similar efficacy, real-world data on sequencing of these therapies have mainly been generated in non-US populations (8, 9). There are limited data available among US populations; a recent study demonstrated longer overall survival (OS) and time to treatment discontinuation (TTD) among sequential regorafenib first (R-T) vs. FTD/TPI first (T-R) (10).

Several recent clinical trials were significant in guiding treatment decisions. The results of the SUNLIGHT phase 3 trial reported improved OS and progression-free survival (PFS) for patients initiating FTD/TPI + bevacizumab compared to FTD/TPI alone (11). Phase 2 ReDOS trial (which compared a regorafenib dose-escalation strategy, defined as an initiation dose of 80 mg/day with a weekly escalation in 40-mg increments to 160 mg/day if no significant drug-related adverse events occur, with the standard dose defined as 160 mg/day for 21 days of a 28-day cycle) found that OS was higher in the dose-escalation arm (12).

Since there is no specific guidance on the preferred order of treatments of interest according to NCCN Guidelines^®^, the aim of this study was to retrospectively assess the duration of therapy and clinical outcomes of R-T and T-R from a real-world perspective (7). This study examined demographic and clinical characteristics, treatment patterns, and clinical effectiveness among patients with mCRC who initiated sequential regorafenib and FTD/TPI ± bevacizumab between first line (1L) and sixth line (6L) and treated between 2015 and 2022 within The US Oncology Network.

## 2 Methods

This was a retrospective observational study of adult patients diagnosed with mCRC treated with sequential regorafenib and FTD/TPI ± bevacizumab between 1L and 6L in The US Oncology Network between September 1, 2015, and November 30, 2022. Patients were followed longitudinally until death or last patient record prior to the end of the study period, May 31, 2023.

Patient and treatment characteristics were assessed descriptively overall and stratified by treatment order. The Kaplan–Meier methods were used for time-to-event endpoints, including real-world overall survival (rwOS), real-world progression-free survival (rwPFS), and real-world time to next treatment (rwTTNT), following sequence. Endpoints were also evaluated using Cox proportional hazards models.

### 2.1 Data source

This study utilized iKnowMed™ (iKM) electronic health record (EHR) data, which is maintained by Ontada. iKM is an EHR system focused on oncology that is implemented across The US Oncology Network, a large community oncology network including over 2,500 providers in more than 600 sites of care across the USA (13). Study data were sourced from the structured fields of iKM EHR data and were supplemented with unstructured data abstracted from patient charts and vital status provided by Datavant.

The iKM database collects records of outpatient visits for patients receiving community-based care. This includes patient demographics (age, race, and gender), clinical details (disease diagnosis and diagnosis stage), and cancer-related treatment details (treatment, treatment dosage, line of therapy, and start and end dates). Datavant includes records of death reported by claims data, obituary records, and the Social Security Administration’s Limited Access Death Master file. If there was a discrepancy between iKM and Datavant vital status data, iKM data were prioritized.

### 2.2 Inclusion and exclusion criteria

The eligibility criteria included patients at least 18 years of age at first diagnosis of CRC who undertook initiation of sequential regorafenib and FTD/TPI between 1L and 6L (index event is earliest initiation date) during the study identification period (September 1, 2015, to November 30, 2022), had at least two visits within The US Oncology Network following index date and during the study observation period (September 1, 2015, to May 31, 2023), and had data accessible for research purposes. The index drug is the first drug initiated in the treatment sequence.

Patients were excluded from the study if there was prior use of regorafenib or FTD/TPI, were enrolled in an interventional clinical trial, or were treated for other documented primary cancers (excluding basal cell carcinoma and squamous cell carcinoma) during the study observation period.

### 2.3 Study outcomes and cohorts

Treatment sequencing, duration, and dosing were assessed. The first treatment following metastatic colorectal cancer diagnosis date was considered 1L treatment. The treatment line was advanced if there was a record of provider-documented progression. Key exceptions were made to the treatment sequencing rules regarding anti-VEGF treatments (bevacizumab, ramucirumab, and ziv-aflibercept) (14). The line of therapy was not advanced when a patient initiated an anti-VEGF treatment, even if the reason for initiation was progression. Thus, the addition of an anti-VEGF to an existing regimen did not result in the start of a new regimen. The duration of therapy was assessed from the first administration date to treatment discontinuation, including treatment interruptions <120 days in length, and did not include days of clinical benefit. Initiation dose, ending dose, and regorafenib dose changes were abstracted from the EHR.

rwOS, rwPFS, and rwTTNT were assessed. rwOS was defined as the interval between the index date and the date of death due to any cause as documented in Datavant or EHR. rwPFS was defined as the interval between the index date and the earliest date of progression or death due to any cause. rwTTNT was defined as the interval between the index date and the treatment following sequence completion. Study outcomes were summarized for the overall population, regorafenib first (R-T), and FTD/TPI ± bevacizumab first (T-R).

### 2.4 Statistical analysis

Descriptive methods were used to assess patient demographics and clinical and treatment characteristics. The Kaplan–Meier methods with 95% confidence intervals (CIs) were used to assess rwOS, rwPFS, and rwTTNT. For rwOS, patients who did not have a date of death documented within the study observation period were censored on the last contact date available. For rwPFS, patients who did not have a date of progression or death within the study observation period were censored at the last contact date available. For rwTTNT, patients who did not initiate a treatment following sequence completion and were still alive at the end of the study observation period were censored at the last contact date available.

## 3 Results

### 3.1 Study population

**Figure 1:**
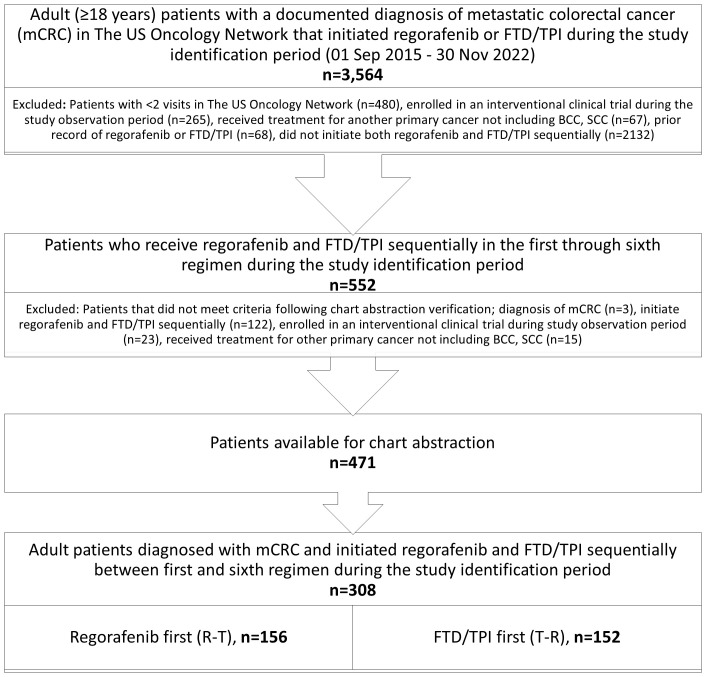
Study population.

After applying inclusion and exclusion criteria, a total of 308 adult patients diagnosed with mCRC that initiated sequential regorafenib and FTD/TPI ± bevacizumab were identified: 156 R-T and 152 T-R. **Figure 1** shows the study population attrition.

### 3.2 Demographics and clinical characteristics

The demographics and clinical characteristics (**Table 1**) at baseline for the overall study population and stratified by treatment order were examined. The demographic characteristics (**Table 1**) were similar across treatment cohorts. Overall, mCRC patients initiating sequential regorafenib and FTD/TPI ± bevacizumab had a mean age of 63 years, and most patients were male (54.5%, n = 168) and Caucasian (66.2%, n = 204). The findings were similar to those of the first treatment. Patients in the R-T cohort had a higher proportion of patients who were overweight (32.1% R-T vs. 30.3% T-R) or obese (29.5% R-T vs. 23.7% T-R).

**Table 1:** Baseline demographic and clinical characteristics by overall and treatment order.

Variable	Overall (N = 308)	R-T (N = 156)	T-R (N = 152)
Age at baseline (years)			
Mean (SD)	63.3 (10.6)	64.1 (10.7)	62.5 (10.5)
Age group at baseline, n (%)			
<65	175 (56.8)	83 (53.2)	92 (60.5)
65+	133 (43.2)	73 (46.8)	60 (39.5)
Gender, n (%)			
Female	140 (45.5)	76 (48.7)	64 (42.1)
Male	168 (54.5)	80 (51.3)	88 (57.9)
Race, n (%)			
Asian	19 (6.2)	8 (5.1)	11 (7.2)
African American	32 (10.4)	14 (9.0)	18 (11.8)
Caucasian	204 (66.2)	106 (67.9)	98 (64.5)
Not documented	39 (12.7)	21 (13.5)	18 (11.8)
Other ^a^	14 (4.5)	7 (4.5)	7 (4.6)
Practice location, n (%)			
Midwest	74 (24.0)	35 (22.4)	39 (25.7)
Northeast	21 (6.8)	9 (5.8)	12 (7.9)
South	114 (37.0)	49 (31.4)	65 (42.8)
West	99 (32.1)	63 (40.4)	36 (23.7)
Initial cancer diagnosis, n (%)			
Colon cancer	215 (69.8)	105 (67.3)	110 (72.4)
Rectal cancer	93 (30.2)	51 (32.7)	42 (27.6)
Stage at initial colorectal cancer diagnosis, n (%) ^b^			
I–II	44 (14.2)	29 (18.6)	15 (9.9)
III	76 (24.7)	32 (20.5)	44 (28.9)
IV	182 (59.1)	90 (57.7)	92 (60.5)
Not documented	6 (1.9)	5 (3.2)	1 (0.7)
Distant metastatic sites, n (%)			
Distant lymph nodes	40 (13.0)	22 (14.1)	18 (11.8)
Liver	201 (65.3)	101 (64.7)	100 (65.8)
Lung	93 (30.2)	47 (30.1)	46 (30.3)
Other ^c^	99 (32.1)	53 (34.0)	46 (30.36)
ECOG at baseline, n (%) ^d^			
0	33 (10.7)	18 (11.5)	15 (9.9)
1	147 (47.7)	71 (45.5)	76 (50.0)
2+	26 (8.4)	16 (10.3)	10 (6.6)
Not documented	102 (33.1)	51 (32.7)	51 (33.6)
BMI category at baseline, n (%) ^d^			
Underweight	<5	<5	<5
Normal	113 (36.7)	51 (32.7)	62 (40.8)
Overweight	96 (31.2)	50 (32.1)	46 (30.3)
Obese	82 (26.6)	46 (29.5)	36 (23.7)
Not documented	13 (4.2)	7 (4.5)	6 (3.9)
KRAS, n (%)			
Positive	139 (45.1)	74 (47.4)	65 (42.8)
Negative	122 (39.6)	56 (35.9)	66 (43.4)

BMI, body mass index; ECOG, Eastern Cooperative Oncology Group performance status scale.

a Native American, Alaska Native, Native Hawaiian or Other Pacific Islander, or other.

b The earliest available diagnosis stages during the study observation period are reported.

c Includes adrenal gland, bone, brain, omentum, peritoneum, pleura, and sites not otherwise specified.

d Assessed within 30 days of index date.

Clinical characteristics (**Table 1**) were also similar across treatment cohorts. Overall, most patients had an initial diagnosis of colon cancer (69.8%, n = 215) and presented with Stage IV disease at the initial diagnosis (59.1%, n = 182). Patients in the T-R cohort had a higher proportion of patients who presented with Stage IVC disease at initial diagnosis (4.2% R-T vs. 11.8% T-R). Most patients had one metastatic site (67.2%, n = 207), and the most common sites were the liver (65.3%, n = 201) and lungs (30.2%, n = 93). Findings were similar by first treatment.

Laboratory results were assessed in the 30 days prior to and following the index date. Overall, the majority of patients had normal alanine transaminase (ALT) (78.9%, n = 243), aspartate aminotransferase (AST) (76.0%, n = 234), total bilirubin (82.5%, n = 254), and serum creatinine (59.4%, n = 183). Findings were similar by first treatment.

### 3.3 Treatment patterns

Most patients included in the R-T cohort (n = 156) initiated index regorafenib in 3L (49.4%, n = 77), 4L (20.5%, n = 32), and 2L (21.2%, n = 33). Similarly, most of the 152 patients in the T-R cohort mostly initiated FTD/TPI in 3L (45.4%, n = 69), 4L (24.3%, n = 37), and 2L (19.1%, n = 29) (**Table 2**). The median (Q1, Q3) duration of therapy of the index drug was 3.0 (1.9, 4.0) months among the R-T cohort and 3.4 (2.6, 5.1) months among the T-R cohort (**Table 3**). The median (Q1, Q3) duration of therapy for the treatment sequence was 7.0 (5.1, 9.8) months overall, with 7.4 (5.2, 10.8) for R-T and 6.7 (4.9, 9.3) for T-R (**Table 3**). Overall, the median (Q1, Q3) time to initiation of regorafenib and FTD/TPI ± bevacizumab from initial colorectal cancer diagnosis and diagnosis of metastatic disease was 31.4 (21.5, 50.7) months and 25.3 (16.7, 35.2) months, respectively.

**Table 2:** Treatment characteristics by overall and treatment order.

Variable	Overall (N = 308)	R-T (N = 156)	T-R (N = 152)
Regorafenib Lot, n (%)			-
1L	4 (2.6)	4 (2.6)	–
2L	33 (21.2)	33 (21.2)	–
3L	77 (49.4)	77 (49.4)	–
4L	32 (20.5)	32 (20.5)	–
5L	10 (6.4)	10 (6.4)	–
FTD/TPI LOT, n (%)			
1L	3 (2.0)	–	3 (2.0)
2L	29 (19.1)	–	29 (19.1)
3L	69 (45.4)	–	69 (45.4)
4L	37 (24.3)	–	37 (24.3)
5L	11 (7.2)	–	11 (7.2)
6L–7L	3 (2.0)	–	3 (2.0)
Number of subsequent lines of therapy, n (%) (following second treatment)			
0	225 (73.1)	104 (66.7)	121 (79.6)
1	54 (17.5)	34 (21.8)	20 (13.2)
2	25 (8.1)	16 (10.3)	9 (5.9)
≥3	4 (1.3)	2 (1.3)	2 (1.3)
Reason for treatment discontinuation of index drug, n (%)			
Progression	262 (85.1)	117 (75.0)	145 (95.4)
AE	43 (14.0)	37 (23.7)	6 (3.9)
Financial/insurance	1 (0.3)	0 (0.00)	1 (0.7)
Other	2 (0.6)	2 (1.3)	0 (0.00)
Treatments prior to index date, n (%)			
Anti-EGFR (cetuximab and panitumumab)	113 (36.7)	53 (34.0)	60 (39.5)
Anti-VEGF monotherapy (bevacizumab, ramucirumab, and ziv-aflibercept)	276 (89.6)	138 (88.5)	138 (90.8)
Patients who recycled doublet therapy prior to index date, n (%)^a^	70 (22.7)	26 (16.7)	44 (28.9)
FOLFOX	35 (11.4)	16 (10.3)	19 (12.5)
FOLFIRI	39 (12.7)	12 (7.7)	27 (17.8)

LOT, line of therapy; R-T, regorafenib first; T-R, FTD/TPI first; FTD/TPI, trifluridine/tipiracil.

a Patients who initiated doublet therapy (FOLFOX or FOLFIRI) more than once prior to index date.

**Table 3:** Treatment duration and dosage by overall and treatment order.

Variable	Overall (N = 308)	R-T (N = 156)	T-R (N = 152)
Treatment duration			
Regorafenib (index) regimen duration (months)			
Patients with available data	–	156	152
Mean (SD)	–	3.5 (2.6)	2.8 (2.2)
Median (IQR)	–	3.0 (1.9, 4.0)	2.3 (1.3, 3.5)
FTD/TPI (index) regimen duration (months)			
Patients with available data	–	156	152
Mean (SD)	–	4.7 (4.6)	4.9 (5.9)
Median (IQR)	–	3.0 (2.0, 5.6)	3.4 (2.6, 5.1)
Sequence duration (months)			
Patients with available data	308	156	152
Mean (SD)	8.5 (6.1)	8.9 (5.6)	8.2 (6.7)
Median (IQR)	7.0 (5.1, 9.8)	7.4 (5.2, 10.8)	6.7 (4.9, 9.3)
Regorafenib dose optimization (REDOS), n (%)			
REDOS ^a^	17 (5.5%)	17 (10.9%)	0
REDOS-probable ^b^	119 (38.6%)	59 (37.8%)	60 (39.5%)
All other dose-adjusted ^c^	51 (16.6%)	20 (12.8%)	31 (20.4%)
Sequence duration (months), among patients utilizing REDOS strategy			
Patients with available data	17	17	0
Mean (SD)	7.6 (4.3)	7.6 (4.3)	–
Median (IQR)	7.4 (4.9, 8.0)	7.4 (4.9, 8.0)	–
Sequence duration (months), among patients not utilizing REDOS strategy			
Patients with available data	291	139	152
Mean (SD)	8.6 (6.2)	9.0 (5.8)	8.2 (6.7)
Median (IQR)	7.0 (5.2, 10.0)	7.4 (5.2, 11.4)	6.7 (4.9, 9.3)

R-T, regorafenib first; T-R, FTD/TPI first; FTD/TPI, trifluridine/tipiracil.

a Patients initiating regorafenib at 80 mg and with an increase in dosage within the first 3 weeks of initiation.

b Patients initiating regorafenib at 80 mg with a) no increase in dosage or b) increase dosage after 3 weeks of initiation.

c Patients with an increase in regorafenib dosage in the first 3 weeks or patients with an initiation dosage <160 mg.

**Figure 2:**
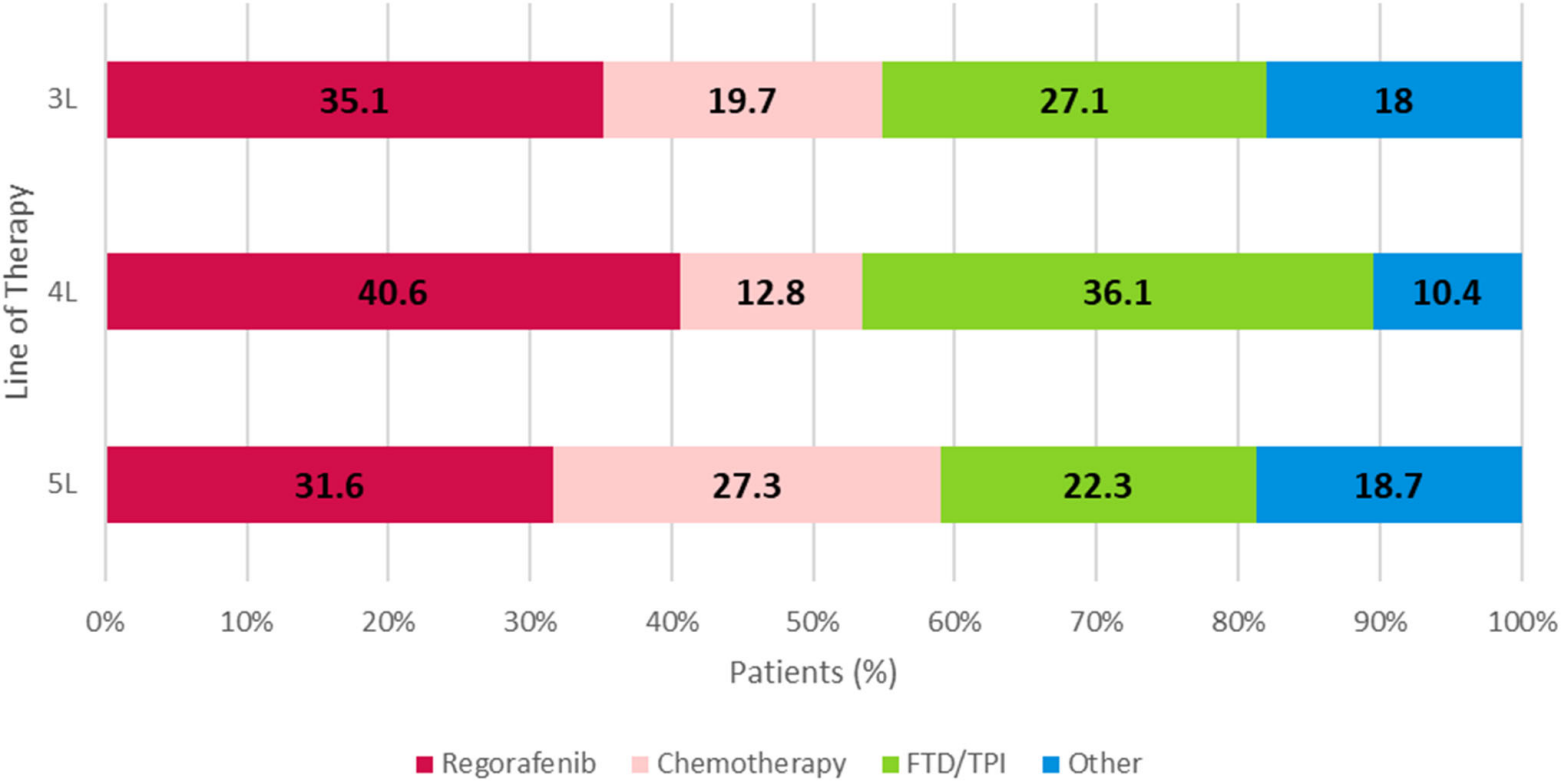
3L, 4L, and 5L overall treatment regimens of metastatic colorectal cancer patients. Chemotherapy: FOLFOXIRI-based, FOLFOX-based, FOLFIRI-based, or capecitabine, fluorouracil, irinotecan, oxaliplatin. Regorafenib: regorafenib-based (monotherapy or in combination with other or FTD/TPI. FTD/TPI: monotherapy. Other: cetuximab and panitumumab, atezolizumab, avelumab, cemiplimab, durvalumab, ipilimumab, nivolumab, pembrolizumab, dostarlimab-gxly, bevacizumab, ramucirumab, ziv-aflibercept, and leucovorin. FTD/TPI, trifluridine/tipiracil.

The treatment received from 1L to 6L was assessed; treatment categories from 3L to 5L are shown in **Figure 2**. Among patients who initiated 4L treatment, regorafenib (40.6%, n=98) and FTD/TPI ± bevacizumab (36.1%, n=87) were the most common regimens initiated.

Overall, the majority of patients initiated a dose-adjusted regorafenib treatment (60.7%, n = 187), defined as ReDOS, ReDOS-probable, or other dose-adjusted. The proportion of patients initiating dose-adjusted regorafenib treatment was similar between the R-T and T-R cohorts (61.5%, n = 96; and 59.9%, n = 91, respectively). However, a higher proportion of physicians were able to successfully implement the ReDOS dosing strategy, defined as patients initiating regorafenib at 80 mg and with an increase in dosage within the first 3 weeks of initiation, among R-T patients (10.9%, n = 17) compared to T-R patients (n = 0). A higher proportion of FTD/TPI ± bevacizumab first patients discontinued treatment due to progression (FTD/TPI ± bevacizumab: 95.4%, n = 145; regorafenib: 75%, n = 117). Overall, many patients used a doublet therapy (FOLFOX or FOLFIRI) more than once prior to the index date (22.7%, n = 70) (**Table 2**).

### 3.4 Outcomes

Overall, the median (Q1,Q3) duration of follow-up time from the index date was 9.9 (6.6, 14.9) months. The median (Q1,Q3) duration of follow-up among patients who initiated R-T and T-R were 11.3 (6.8, 16.1) months and 8.9 (6.5, 13.7) months, respectively. rwOS, rwPFS, and rwTTNT were measured overall and by treatment order.

**Figure 3:**
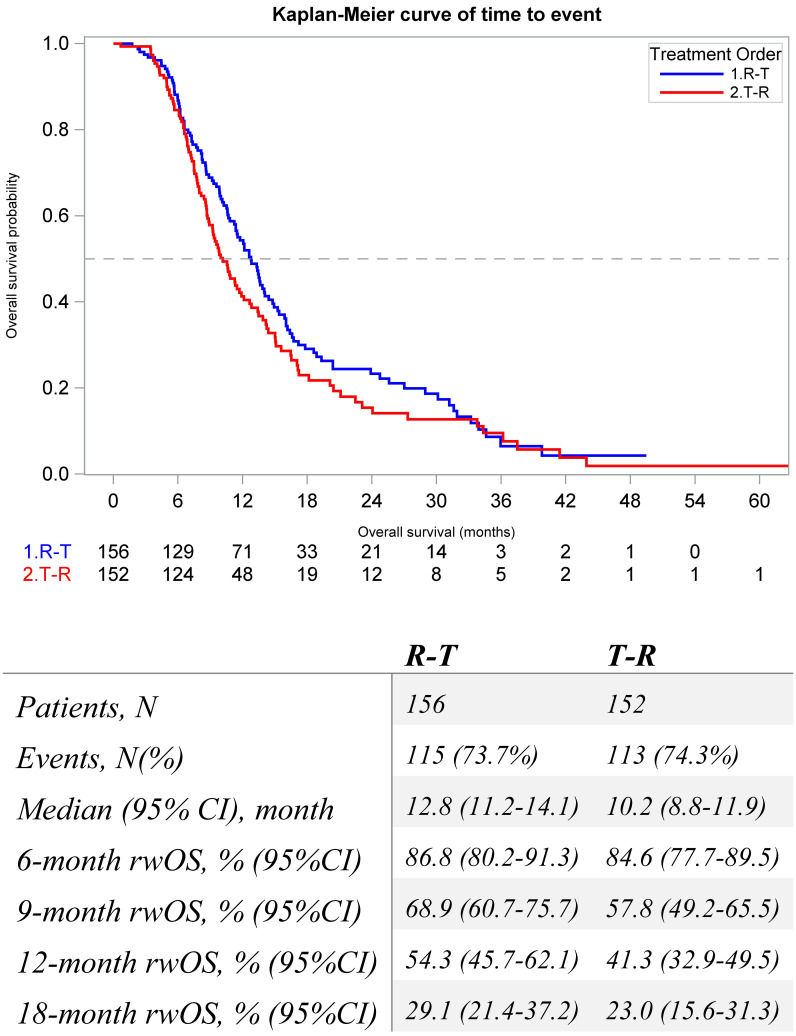
Kaplan-Meier Estimates for real-world overall survival of metastatic colorectal patients that initiate sequential regorafenib and FTD/TPI between 1L and 6L by treatment order.

The median (95% CI) rwOS was numerically longer among the R-T cohort compared to the T-R cohort (12.8 [11.2, 14.1] vs. 10.2 [8.8, 11.9] months) (**Figure 3**). Factors associated with rwOS were analyzed with multivariable Cox analysis, and the following covariates were associated with significantly poorer survival: stage at diagnosis Stage IV (HR = 1.5, p = 0.003) and prior anti-VEGF treatment (HR = 1.79, p = 0.017). Index treatment was not significantly associated with rwOS (HR = 1.2, p = 0.2) (**Supplementary Table 1**).

The median (95% CI) rwPFS was 3.4 (3.0, 3.6) and 3.4 (3.0, 3.7) months for both the R-T and T-R cohorts, respectively. After adjusting for clinically and statistically significant factors, there was no significant difference (HR = 0.9, p = 0.4).

**Figure 4:**
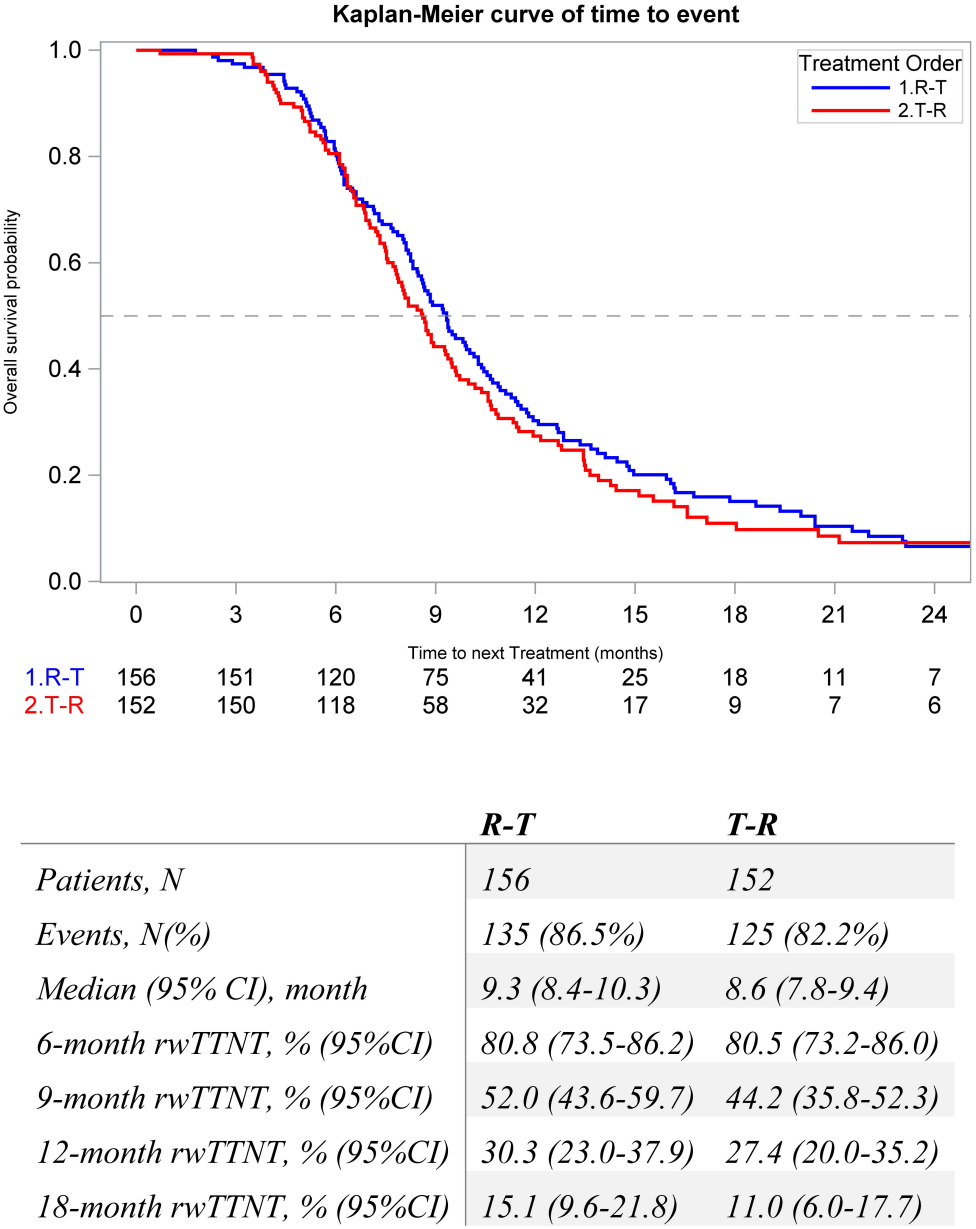
Kaplan-Meier Estimates for real-world time to next treatment in metastatic colorectal patients that initiate sequential regorafenib and FTD/TPI between 1L and 6L by treatment order.

The median (95% CI) rwTTNT following sequence was numerically longer among the R-T cohort compared to the T-R cohort (9.3 [8.4, 10.3] vs. 8.6 [7.8, 9.4] months) (**Figure 4**). Factors associated with rwTTNT were analyzed with multivariable Cox analysis, and patients with Stage IV at diagnosis had poorer outcomes (HR = 1.40, p = 0.01). Index treatment was not significantly associated with rwTTNT (HR = 1.1, p = 0.6) (**Supplementary Table 2**).

## 4 Discussion

This study assessed the patient characteristics, treatment characteristics, and outcomes among patients initiating R-T, T-R, and overall. Patients who initiated R-T had a numerically longer duration of therapy, rwOS, and rwTTNT. While this study showed a potential trend toward improved overall survival and time to next treatment for R-T patients, the findings should be interpreted within the framework of other studies with similar directional results. Treatment decisions should be tailored to individual patient profiles in order to maximize the clinical benefit of life-prolonging medication. The unadjusted rwOS results of our study align with a previous real-world study completed in US-based patients using the Flatiron Health electronic health records, which also demonstrated that R-T patients appeared to have a numerically longer rwOS than T-R patients (13.1 vs. 11.5 months among 3L; 11.6 vs. 10.3 among 4L) (10).

The sample of mCRC patients in our study had demographic and clinical characteristics consistent with other real-world studies. The National Cancer Institute’s Surveillance, Epidemiology, and End Results Program (SEER) reported that mCRC patients have a median age of 66 years, and the disease is more common in men than women (15). The median age for our study population was 63 years, and 54.5% of patients were male. The patient profile also matched those of studies conducted in other US-based real-world studies utilizing the Flatiron and Optum databases (10, 16). The median treatment duration of index regorafenib aligned with previous studies, including the real-world CORRELATE study, which reported a median (range) treatment duration of 2.5 (0.03–29.5) months (17).

Another real-world study conducted in Italian cancer centers had similar findings that demonstrated a statistically significant difference in unadjusted median rwOS (15.9 vs. 13.9 months, p = 0.0194, for R-T vs. T-R, respectively) and unadjusted median rwPFS (11.2 vs. 8.8 months, p = 0.0005, for R-T vs. T-R, respectively) (18). Patients were required to have a RAS mutation status and progression following exposure to at least two prior regimens of standard chemotherapy, anti-VEGF, or EGFR antibodies. Additionally, our study is strengthened by the use of multivariable Cox regression models.

Since the ReDOS trial, a phase II dose optimization trial, demonstrated improved outcomes for the dose-escalation cohort, the NCCN Guidelines updated its recommendations on March 14, 2018, by recommending the ReDOS dosing strategy (7, 12, 16). As a result, many patients (60.7%, n = 187) in our study population initiated a dose-adjusted regorafenib treatment. Prior real-world studies have determined that patients receiving flexible dosing regimens had a longer duration of therapy compared to patients with standard dosing, even in the presence of adverse prognostic factors (19). Our study was aligned with those results and demonstrated that patients with ReDOS-probable dosing had a median (IQR) sequence duration of therapy of 7.4 (4.9, 8.0) months, and patients without ReDOS had a median (IQR) duration of 7.0 (5.2, 10.0) months.

The FDA approved FTD/TPI + bevacizumab in August 2023 for third-line treatment for mCRC patients previously treated with fluoropyrimidine, oxaliplatin-, and irinotecan-based chemotherapy; a VEGF inhibitor; and an EGFR inhibitor, if they have RAS wild-type disease. NCCN Guidelines updated their treatment guidelines, suggesting a preference for FTD/TPI + bevacizumab over FTD/TPI monotherapy (7). European Society for Medical Oncology (ESMO) Metastatic Colorectal Cancer Living Guidelines updated their treatment recommendations in February 2024 (20). Our study had a limited number of eligible patients initiating FTD/TPI + bevacizumab (16 patients each in both R-T and T-R cohorts) as the study identification period ended in November 2022, prior to the publication of SUNLIGHT Phase 3 trial data results. While the subgroup of patients who initiated FTD/TPI + bevacizumab may have had improved outcomes, similar patient characteristics and small sample size likely had minimal impact on results in this study.

This study shows that overall survival in mCRC patients requiring later lines of therapy is approximately 12 months. Therapies approved for later lines, such as regorafenib and FTD/TPI, are key in prolonging patient survival, and their benefits in the real-world setting support results seen from pivotal clinical Phase 3 trials (6, 21). This study provides insight into the real-world treatment landscape of patients treated with sequential R-T and T-R and demonstrates that patients initiating R-T and T-R have similar outcomes. Access to all 3L approved therapies should be maintained so physicians may tailor therapies based on patient profiles. Future research should focus on increasing cohort sample size and utilizing more contemporary data following the FDA approval of FTD/TPI + bevacizumab and analyze outcomes between patients receiving sequential regorafenib and FTD/TPI + bevacizumab. This study also demonstrated opportunities to improve care. In the R-T and T-R cohorts, 16.7% and 28.9% of patients, respectively, recycled a cytotoxic doublet prior to the index date. Clinical evidence suggests that chemotherapy recycling should be used as true salvage therapy after standard-of-care therapies have been exhausted and is a less supported strategy to improve overall survival. As a result, treatments of interest (regorafenib and FTD/TPI ± bevacizumab) should be initiated sooner while patients have better performance status and potential for benefit (22). Provider education on strategies to optimize care for patients is necessary.

As a retrospective assessment of community-based oncology clinics, these findings provide real-world evidence to describe the treatment landscape and clinical outcomes of patients with sequential treatment of regorafenib and FTD/TPI ± bevacizumab and are more representative of the general population than clinical trials. Multivariable analyses for rwOS, rwPFS, and rwTTNT were conducted to increase the robustness of this study. Chart abstraction allowed for confirmation that both treatments were initiated sequentially and allowed for granular data capture of dose changes.

### 4.1 Limitations

This study should be considered in the context of the limitations of the data source and study design. EHR data are recorded for clinical care and not for research purposes. Variables may not be available, such as disease sidedness, or complete across the entire study population. Data regarding patient care outside of The US Oncology Network are not recorded uniformly in chart notes across patients. The lack of uniformly available patient characteristics may induce selection bias, but adjusted analyses of outcomes were conducted to account for differences. Patient demographics and clinical characteristics were assessed at baseline; changes during the follow-up period were not evaluated or reported and may impact outcomes. Results may not be generalizable to the US population diagnosed with metastatic colorectal cancer due to the use of evidence-based guidelines in The US Oncology Network. Oral medications, including regorafenib and FTD/TPI ± bevacizumab, were assumed to be taken as prescribed.

A higher proportion of R-T patients followed the ReDOS strategy, which may have had minimal impact on the results. There was a similar proportion of patients following the ReDOS-probable sequence. It is also important to acknowledge that the study’s eligibility criteria required patients to have adequate health to undergo both treatments sequentially, introducing survivorship bias and limiting the generalizability to a specific subset of patients.

### 4.2 Conclusion

This study provides insight into real-world outcomes and treatment patterns among patients initiating sequential regorafenib and FTD/TPI ± bevacizumab in a US community oncology setting. Initiating therapy R-T ± bevacizumab resulted in numerically longer rwOS and rwTTNT, although there were no statistically significant differences. Sequential use of regorafenib and FTD/TPI ± bevacizumab or vice versa may provide similar benefits to patients. Providing access to regorafenib and FTD/TPI ± bevacizumab is critical to maximizing patient benefit and optimizing patient care in advanced stages of mCRC.

## Data Availability

The health data used to support the findings of this study are restricted by The US Oncology Research Institutional Review Board in order to protect patient privacy. For this reason, data used to support the findings of this study have not been made available.
