# Supplementary material for: Real-world treatment patterns and outcomes among patients initiating sequential regorafenib and trifluridine/tipiracil ± bevacizumab in patients with metastatic colorectal cancer in a US community setting (SEQRT2)
Source: Front Oncol. 2025 Jun 11;15:1591245. doi: 10.3389/fonc.2025.1591245 (PMC12188609; doi:10.3389/fonc.2025.1591245)
Supplement: Supplementary file 1 [file Table1.docx]

**Supplementary Table 1. Multivariate Cox Regression model on OS**

| Covariate | Level | Total | Censored | Event | HR (95% CI) | Effect | Type3 |
| --- | --- | --- | --- | --- | --- | --- | --- |
| Age | < 65 (reference) | 175 | 47 | 128 |  |  | 0.4811 |
|  | 65 + | 133 | 33 | 100 | 1.104 (0.838, 1.453) | 0.4811 |  |
| Gender | Female (reference) | 140 | 30 | 110 |  |  | 0.3777 |
|  | Male | 168 | 50 | 118 | 1.139 (0.853, 1.52) | 0.3777 |  |
| Stage at diagnosis | Stage 0-III (reference) | 120 | 38 | 82 |  |  | **0.0123** |
|  | Stage IV | 182 | 39 | 143 | 1.523 (1.153, 2.012) | **0.003** |  |
|  | Not documented | 6 | 3 | 3 | 1.131 (0.213, 5.997) | 0.8849 |  |
| ECOG | 0 (reference) | 33 | 8 | 25 |  |  | **0.0487** |
|  | 1 | 147 | 34 | 113 | 1.106 (0.729, 1.678) | 0.6358 |  |
|  | 2+ | 26 | 3 | 23 | 1.764 (0.978, 3.184) | 0.0595 |  |
|  | Not documented | 102 | 35 | 67 | 0.869 (0.566, 1.333) | 0.52 |  |
| Prior anti-VEGF | No (reference) | 32 | 14 | 18 |  |  | **0.017** |
|  | Yes | 276 | 66 | 210 | 1.786 (1.109, 2.877) | **0.017** |  |
| Index treatment | Regorafenib (reference) | 156 | 41 | 115 |  |  | 0.2006 |
|  | FTD/TPI | 152 | 39 | 113 | 1.198 (0.908, 1.58) | 0.2006 |  |

The following covariates were considered for inclusion in the model: age, gender, stage at diagnosis, ECOG in the 30 days prior to index date, ECOG in the +/- 30 days of index date, KRAS mutation, prior anti-VEGF treatment, line of therapy, regorafenib dosing, and index treatment.

**Supplementary Table 2. Multivariate Cox regression on TTNT**

| Covariate | Level | Total | Censored | Event | HR (95% CI) | Effect | Type3 |
| --- | --- | --- | --- | --- | --- | --- | --- |
| Age | < 65 (reference) | 175 | 28 | 147 |  |  | 0.6238 |
|  | 65 + | 133 | 20 | 113 | 1.066 (0.826, 1.374) | 0.6238 |  |
| Gender | Female (reference) | 140 | 20 | 120 |  |  | 0.2689 |
|  | Male | 168 | 28 | 140 | 1.157 (0.894, 1.497) | 0.2689 |  |
| Stage at diagnosis | Stage 0-III (reference) | 120 | 21 | 99 |  |  | **0.0421** |
|  | Stage IV | 182 | 25 | 157 | 1.395(1.074, 1.811) | **0.0125** |  |
|  | Not documented | 6 | 2 | 4 | 0.925 (0.171, 4.987) | 0.9274 |  |
| Line of therapy | 3L or 4L (reference) | 215 | 31 | 184 |  |  | 0.1423 |
|  | 1L, 2L, 5L, 6L, 7L | 93 | 17 | 76 | 0.822 (0.632, 1.068) | 0.1423 |  |
| Index treatment | Regorafenib (reference) | 156 | 21 | 135 |  |  | 0.6213 |
|  | FTD/TPI | 152 | 27 | 125 | 1.066 (0.827, 1.373) | 0.6213 |  |

The following covariates were considered for inclusion in the model: age, gender, stage at diagnosis, ECOG in the 30 days prior to index date, ECOG in the +/- 30 days of index date, KRAS mutation, prior anti-VEGF treatment, line of therapy, regorafenib dosing, and index treatment.
